# TLR3-Induced Maturation of Murine Dendritic Cells Regulates CTL Responses by Modulating PD-L1 Trafficking

**DOI:** 10.1371/journal.pone.0167057

**Published:** 2016-12-02

**Authors:** Aditi Varthaman, Hélène D. Moreau, Mathieu Maurin, Philippe Benaroch

**Affiliations:** Institut Curie, PSL Research University, INSERM, U 932, Paris, France; COMUE Sorbonne Universites, FRANCE

## Abstract

Targeting TLR3 through formulations of polyI:C is widely studied as an adjuvant in cancer immunotherapy. The efficacy of such targeting has been shown to increase in combination with anti-PD-L1 treatment. Nevertheless, the mechanistic details of the effect of polyI:C on DC maturation and the impact on T-DC interactions upon PD-L1 blockade is largely unknown. Here we found that although DC treatment with polyI:C induced a potent inflammatory response including the production of type I interferon, polyI:C treatment of DCs impaired activation of peptide specific CD8^+^ T cells mainly due to PD-L1. Interestingly, we found that PD-L1 trafficking to the cell surface is regulated in two waves in polyI:C-treated DCs. One induced upon overnight treatment and a second more rapid one, specific to polyI:C treatment, was induced upon CD40 signaling leading to a further increase in surface PD-L1 in DCs. The polyI:C-induced cell surface PD-L1 reduced the times of contact between DCs and T cells, potentially accounting for limited T cell activation. Our results reveal a novel CD40-dependent regulation of PD-L1 trafficking induced upon TLR3 signaling that dictates its inhibitory activity. These results provide a mechanistic framework to understand the efficacy of anti-PD-L1 cancer immunotherapy combined with TLR agonists.

## Introduction

The pathogen recognition receptor, Toll-like receptor 3 [1] recognizes double-stranded RNA (dsRNA) of certain viruses to induce a potent innate immune response crucial for pathogen control [2–5]. Interestingly, several human tumours express high levels of TLR3 [6] that is being targeted in immunotherapeutic protocols to initiate both innate and adaptive immune responses. PolyI:C, a synthetic dsRNA mimetic and its formulations have shown promising results when administered alone or in combination with other ligands as adjuvants in immunotherapy in both human cancers and in murine tumour models [7, 8]. Two main characteristics of TLR3 signalling make it an ideal target in immunotherapy: i. it induces a strong type I interferon response that exhibits anti-tumoral potential [9], ii. TLR3 is preferentially expressed in cross-presenting DCs and promotes cross-priming of endogenous antigens thereby inducing strong CD8^+^ T cell responses [10]. Thus, polyI:C treatment might not only target TLR3 in tumour cells and induce an anti-tumour type I interferon-rich environment or tumour apoptosis [11] but will also target the maturation and antigen presentation of DCs specialised in the cross-presentation of tumour-associated antigens. The wide expression of TLR3 on macrophages and even on stromal cells that surround the tumour suggests an additional response from these cells upon polyI:C administration that has not yet been clearly elucidated [6, 8].

Despite the numerous studies in mice showing the efficacy of polyI:C as adjuvants *in vivo* [12], there are several instances where polyI:C might be inefficient for the induction of a strong CTL response. Phase II clinical trials using polyI:C in human tumours have also shown mixed results. Interestingly, administration of polyI:C at the same time as the antigen leads to a potent adaptive immune response whereas pre-sensitization with TLR3 ligands leads to inefficient immune responses [13–18]. The timing and route of the administration of polyI:C seems to impact on the efficiency of the CTL response induced [19, 20]. Furthermore, polyI:C has been notoriously shown to induce the expression of PD-L1, a widely expressed cell surface molecule that inhibits T cell responses through PD-1 [15]. Indeed, recent studies show an unprecedented efficacy of a combined treatment with polyI:C and anti-PD-L1 blocking antibodies [15, 21, 22, 23].

It is important to understand the adaptive immune response induced by TLR3 ligands in order to comprehend and improve outcomes of immunotherapeutic strategies. Here, we assessed the impact of polyI:C-induced maturation of DCs on a naive CD8^+^ T cell response *in vitro*. Surprisingly, we observed that the presentation of a minimal OVA peptide to naive OT1 T cells was relatively inefficient when DCs were matured with polyI:C as compared to LPS, a TLR4 ligand that did not induce type I interferon in our system. Blockade of PD-L1 restored OT1 T cell proliferation in polyI:C-matured DCs but did not affect LPS-matured DC cultures. Our data further suggest that interaction with T cells via CD40 leads to selective trafficking of PD-L1 to the cell surface in polyI:C-matured DCs potentially explaining the difference in susceptibility of polyI:C and LPS-matured DCs to PD-L1 blockade. Importantly, our results also shed light on a mechanism of synergistic action of polyI:C and anti-PD-L1 anti-tumoral immunotherapy.

## Materials and Methods

### Mice and cells

C57BL/6J mice were obtained from Charles River. TLR3 knockout mice were a kind gift from Dr. Monique Lafon (Institut Pasteur, France) and have been described in [1]. PD-L1 knockout mice were obtained from Dr. Heinz Wiendl (University of Münster). OT-1 RAG-KO and *Ubi*-GFP OT1 mice were bred in the Institut Curie animal facility. *Ubi*-GFP OT1 cells were derived by crossing OT-1 RAG1-KO transgenic mice to *Ubi*-GFP mice expressing the GFP protein under the control of the ubiquitin C promoter. Bone marrow-derived DCs (BMDCs) were obtained by differentiating bone marrows with granulocyte/macrophage colony-stimulating factor (40 ng/mL)–containing medium (IMDM with 10% heat-inactivated serum, penicillin/streptomycin, 2mM L-glutamine, 50μM ß-mercaptoethanol) during 10 days. Supernatant from J558 plasmacytoma cells was used as GM-CSF source. B3Z cells specific for the SIINFEKL (OVA257–264/Kb) peptide of OVA that express β-galactosidase under the control of the IL-2 promoter were used for the cross-presentation assay. The present experiments, which used mouse strains exhibiting non-harmful phenotypes, did not require a project authorization and benefited from guidance of the Animal Welfare Body, Research Centre, Institut Curie. No specific approval was obtained from the animal welfare body for this research. Mice were sacrificed by cervical dislocation. All animal procedures and breeding were in accordance with the guidelines and regulations of the French Veterinary Department. The mice were bred and housed in an accredited animal facility (C75-05-18).

### Reagents

Low molecular weight polyI:C (referred to as polyI:C, 0.2-1kb in size) was obtained from Invivogen. LPS (from *Salmonella enterica enterica*, serovar Typhimurium) and celastrol were from Sigma-Aldrich. Anti-PD-L1 antibody (clone 10F.9G2) and the rat isotype control antibody were from BioXcell and used for blocking experiments at 20 μg/mL. Fluorochrome-labelled antibodies to PD-L1 (clone MIH5), PD-1 (clone J43), CD86 (clone GL1), CD40 (clone HM40-3), H2K^b^ (clone AF6-88.5), CD80 (clone 16-10A1), CD40L (clone MR1) and the isotype controls were from BD bioscience. Mouse recombinant CD40L-Fc chimera was from R&D systems.

### Cross presentation of bead-bound OVA

Amine-modified polystyrene beads (Polysciences) were pre-activated with 8% (vol/vol) glutaraldehyde for 3 h at room temperature. Beads were incubated with 20 mg/mL OVA or BSA to different percentages of OVA (Worthington Biochemicals) (100%, 50%, 25% or 0% OVA) on a rotating wheel overnight at 4°C. The next day, the beads were quenched in PBS containing 0.5 M glycine for 30 min before assays with cells. DCs were incubated with bead-bound OVA for 3 h and washed twice with PBS 2% BSA. B3Z cells were added at a 1:1 ratio to the DCs and incubated for 16 h. Beta-galactosidase activity was measured using a chemiluminescent 1,2-dioxetane substrate (Applied Biosystems).

### Bead-bound OVA degradation assay

Cytometric analysis of OVA-degradation in the phagosome has been described in detail elsewhere [24]. Briefly, beads were prepared as above with 100% OVA coating. DCs were pulsed with the bead-bound OVA for 30 min at 18°C, washed in ice cold PBS. Fetal calf serum (FCS) flotation was performed 3 times by overlaying the cells on 3 mL of fetal calf serum and centrifuging at 900rpm to remove non-internalised beads. Cells were chased at 37°C in CO2-independent medium for the 30, 60, 90 or 120 min, resuspended in homogenization buffer (8% sucrose in PBS, 3 mM imidazole, 1 mM dithiothreitol) and disrupted mechanically with 2 mL syringes and 22-gauge needles (Terumo Medical). After centrifugation, the post-nuclear supernatant was transferred to 96-well conical microplates followed by labelling on ice with a rabbit polyclonal antibody against OVA (Sigma) and an Alexa647-labelled secondary antibody and analysed using a FACSCalibur flow cytometer.

### OT1 T cell proliferation assay and antibody blocking

DCs (10^5^) were activated with polyI:C (25 μg/mL unless indicated otherwise) or LPS (10 ng/mL unless indicated otherwise) for 20 hours and then washed 3 times with PBS 0.2% BSA. They were incubated with different concentrations of soluble OVA peptide (SIINFEKL) diluted in PBS 0.2% BSA for 3 h at 37°C followed by 3 washes with PBS 0.2% BSA. When antibody blocking was performed, the isotype control or anti-PD-L1 antibodies were added at 20 μg/mL for 1 hour on ice. The cells were washed 3 times with PBS 0.2% BSA before adding 10^5^ CFSE-labelled OT-1 splenocytes prepared by staining with 10μM CFSE (Invitrogen) for 10 min. Three days after co-culture, the number of total CFSE high and low-positive cells were counted with the help of 10μm counting beads (Biovalley) using an Accuri flow cytometer.

### Cytokine assay

Soluble cytokines were measured in the supernatant of DCs activated with polyI:C (25 μg/mL) or LPS (10 ng/mL) for 20 h using a bead-based immunoassay (Flowcytomix technology, eBioscience). Fluorescence was read using an LSRII flow cytometer (BD Bioscience). IFNα and IFNβ were dosed using the ELISA kits from PBL interferon source.

### Immunofluorescence

DCs were plated on glass coverslips in a 24 well plate and activated with nothing, polyI:C (25 μg/mL) or LPS (10 ng/mL). After 16 hrs, recombinant CD40L-Fc chimera (500 ng/mL) was added to the cells for different periods of time. The cells were then fixed with 4% paraformaldehyde for 20 min and stained for PD-L1 in PBS containing 0.5% saponin and 2% BSA. Coverslips were mounted in mounting medium containing DAPI and samples were imaged on a Nikon Ti Inverted Microscope fitted with a confocal A1R system, using a 60x oil immersion objective with a numerical aperture of 1.4.

### Imaging of DC-T cell contact times

On day 10 of culture, DCs from C57BL/6 and PDL-1 KO mice were seeded at 2.10^5^ cells/mL and activated with nothing, polyI:C (25 μg/mL) or LPS (10 ng/mL. On day 11, DCs were loaded with 4 pg/mL of SIINFEKL peptide for 3 h. Splenic *Ubi*-GFP OT-I CD8^+^ T cells, pre-activated for 48 h with coated anti-CD3 antibody (10 μg/mL) and soluble anti-CD28 antibody (1 μg/mL), were added to a DC:T cell ratio of 1:5 or 1:10. Imaging was started immediately, and images were acquired every minute for 12 h, 4 fields per condition. Imaging was performed on an epifluorescence video-microscope Nikon TiE, equipped with a cooled CCD camera (HQ2, Photometrics), a 10X objective, and an environmental chamber controlled for temperature, humidity and CO_2_. Contact times were manually measured using ImageJ software. All T cells present in the fields at 30 min after the beginning of the recording were considered and tracked for the measurement of the contact times with DCs. Since a lot of contacts were longer than the duration of the recording we first analysed the percentage of long contacts (>600 min) as compared to shorter ones (<600 mins). Z-test was used to compute the significance of the differences between all conditions.

### Statistical analysis

All statistical analysis was performed using a two-way ANOVA test followed by Bonferroni or Tukey posttests to determine significance between treatments in Graphpad (Prism).

## Results

### Cross-presentation of bead-bound antigen is drastically reduced in DCs pre-treated with polyI:C

Conventional high molecular weight polyI:C not only triggers TLR3 but also MDA-5 signalling, a cytosolic RNA sensor [25], in bone marrow-derived DCs. To selectively analyse the effect of TLR3 signalling in the ability of DCs to cross-present antigen, we checked that low molecular weight polyI:C induced signalling only through TLR3 in our setting (S1 Fig), and used it throughout the study.

**Fig 1:**
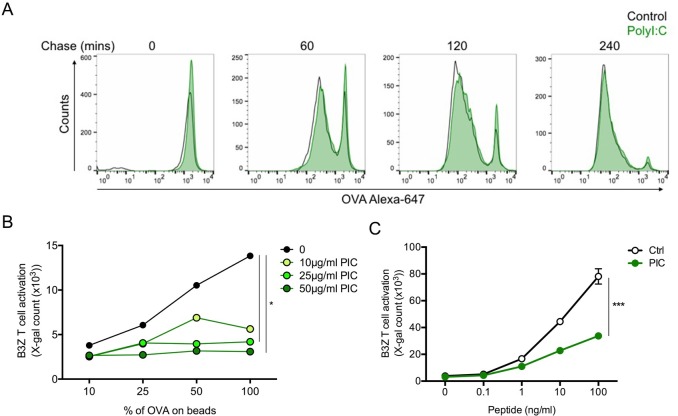
PolyI:C pre-treatment impairs the capacity of DCs to cross-present antigen. (**A**) Degradation of bead-bound OVA was measured in DCs treated with nothing (Control) or polyI:C (25 μg/mL). Pre-treated DCs were pulsed with bead-bound OVA for 30 min at 18°C and chased for 0, 60, 120 or 240 min. The amount of OVA present on internalised beads was evaluated using an anti-OVA polyclonal antibody by FACS. (**B**) Cross-presentation of bead-bound OVA by DCs pre-treated with different concentrations of polyI:C for 20 h. After incubation with bead-bound OVA, cells were washed and incubated with B3Z T cells at a 1:1 ratio for 20 h. Proliferation was measured by detection of ß-galactosidase activity in the cells. (**C**) Presentation of the soluble OVA peptide. DCs were treated with nothing (Ctrl) or polyI:C (25 μg/mL) for 20 h and then loaded with different concentrations of the SIINFEKL peptide for 3 h, washed and incubated with B3Z T cells at a 1:1 ratio for 20 h. Proliferation of B3Z T cells was evaluated by detection of ß-galactosidase activity in the cells. Error bars represent SEM. ** p value < 0.001. Data are representative of 4 independent experiments.

In order to cross-present antigen, DCs uptake proteins into the endocytic pathway where they sample the antigen upon degradation to present MHC class I-loaded peptides to T cells. In order to address the effect of polyI:C on the cross-presentation pathway, we first exposed DCs for 20 h to polyI:C and analysed the capacity of DCs to degrade beads-associated OVA. We found that polyI:C treatment did not affect the ability of DCs to degrade bead-associated OVA (Fig 1A) suggesting that the antigen processing machinery was not affected. However, the ability of polyI:C-matured DCs to cross-present bead-bound OVA to B3Z, an OVA-specific CD8^+^ T cell clone, was significantly reduced upon pre-treatment with polyI:C in a dose-dependent manner (Fig 1B). Surprisingly, we found that the presentation of the soluble OVA peptide was also diminished (Fig 1C) although MHC class I, and the co-stimulation markers CD86, CD80 and CD40 levels increased on the DC surface (S2 Fig). The lower T cell proliferation in response to the soluble OVA peptide, that does not need internalisation or processing to be presented, suggested a second mechanism independent of antigen processing that lowered the ability of DCs to present antigen to B3Z CD8^+^ T cells.

### PolyI:C pre-treatment of DCs is inefficient at inducing a naive CTL response

**Fig 2:**
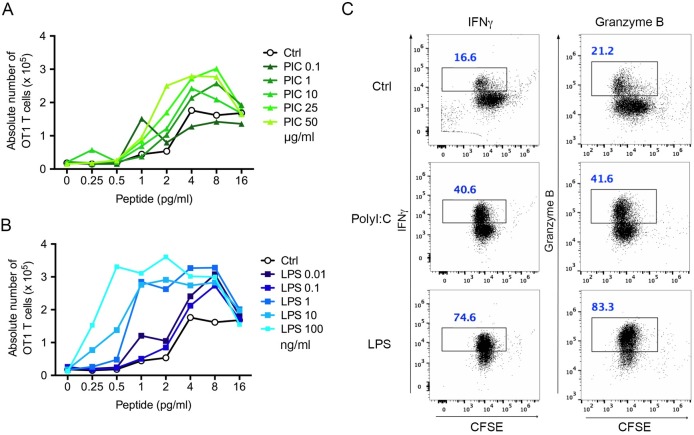
PolyI:C pre-treatment impedes the capacity of DCs to elicit an antigen-specific cytotoxic T cell response. (**A-B**) DCs were either left non-treated (Ctrl) or treated with different concentrations of polyI:C or LPS as indicated for 20 h, washed, loaded with different doses of SIINFEKL peptide and incubated with CFSE-labelled OT1 CD8^+^ T cells at a 1:1 ratio. Three days later, the number of total CFSE-high and low positive T cells were enumerated by FACS-based cell counting. Data are presented as an absolute number of OT1 T cells present in each well. (**C**) OT1 T cells co-cultured for 3 days with polyI:C (25 μg/mL) or LPS (10 ng/ml) pre-treated DCs loaded with 4 pg/mL of SIINFEKL peptide were permeabilized and stained for IFNγ and Granzyme B. Numbers in the windows indicate the percentage of positive cells among total T cells in the well. The figures are representative of 2 independent experiments.

To extend our findings to primary T cells, we performed similar experiments with primary naive OT1 CD8^+^ T cells. Pre-treatment of DCs with polyI:C marginally increased the ability of DCs (loaded with increasing doses of peptide) to induce OT1 T cell proliferation as compared to non-treated DCs (Fig 2A). Moreover, increasing doses of polyI:C did not significantly increase T cell proliferation though the expression of co-stimulatory molecules and MHC I showed a polyI:C dose-dependent increase on the cell surface (S2 Fig). In contrast, LPS pre-treated DCs induced an important LPS dose-dependent increase in OT1 T cell proliferation (Fig 2B). Phenotypic analysis of the OT1 response indicated that polyI:C-matured DCs induced lower frequencies of IFNγ and granzyme B-secreting CD8^+^ T cells than LPS-matured DCs (Fig 2C). The observation that increasing doses of polyI:C was unable to induce higher OT1 T cell proliferation suggested that an active mechanism was in play to restrict T cell responses.

### PD-L1 induced on DCs by polyI:C treatment restricts CD8^+^ T cell proliferation

**Fig 3:**
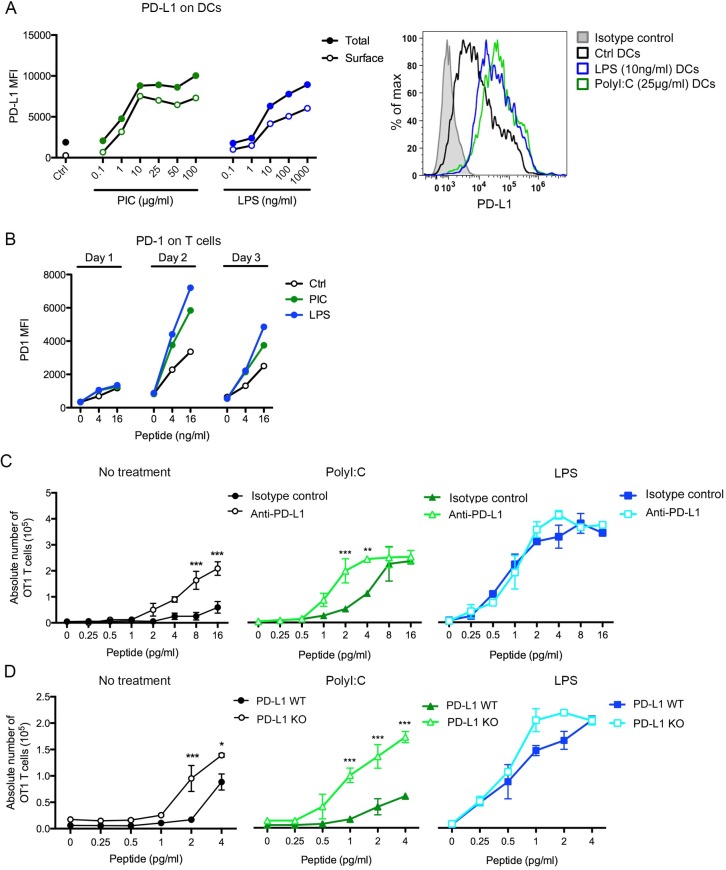
PolyI:C pre-treatment of DCs induces a PD-L1-dependent inhibition of CD8^+^ T cell responses. (**A**) Left: DCs treated with the different doses of TLR ligand for 20 h were stained with an anti-PD-L1 antibody with (total) or without permabilization (surface) and analysed by flow cytometry. Right: FACs profiles of PD-L1 expression on DCs treated with the indicated concentrations of PolyI:C or LPS for 16 h (MFI of Isotype control: 1033 (only one isotype control is shown for clarity, isotypes on all DCs had similar values), Ctrl non-treated DCs: 8504, LPS-pre-treated DCs: 36614, polyI:C pre-treated DCs: 43123). (**B**) MFI of PD-1 on OT1 T cells co-cultured for 1, 2 or 3 days with nothing (ctrl), polyI:C (PIC, 25 μg/ml) or LPS (10 ng/ml)-treated DCs loaded with 0, 4 or 16 pg/mL of SIINFEKL peptide. T cells were gated on their forward and side scatter profile. (**C**) DCs left untreated (ctrl, left) or treated for 20 h with polyI:C (25 μg/mL, middle, green lines) or LPS (10 ng/ml), right, blue lines) were loaded with different concentrations of the SIINFEKL peptide. They were then pre-incubated for 1 h with an anti-PD-L1 antibody or an isotype control antibody (lighter lines) before incubation with OT1 T cells at a 1:1 ratio. 3 days later, the numbers of T cells in the well were enumerated using flow cytometry. (**D**) DCs from wild type mice (WT) or PD-L1 knockout mice (PD-L1 KO) were stimulated or not with polyI:C (25 μg/mL) or LPS (10 ng/ml), loaded with the SIINFEKL peptide and incubated with OT1 T cells at a 1:1 ratio. 3 days later, the numbers of T cells in the well were enumerated using flow cytometry. Figures are representative of 3 independent experiments. *** represents p<0.001, ** p<0,01, * p<0,05 by two-way anova test. Error bars represent SEM.

During activation, DCs up-regulate inhibitory molecules such as PD-L1 that can bind PD-1 on activated T cells and inhibit T cell activation through its ITIM motif [26]. PolyI:C and LPS treatments induced an increase in both intracellular and extracellular levels of PD-L1 with polyI:C inducing slight but consistently higher levels than LPS (Fig 3A). OT1 T cells co-cultured with polyI:C and LPS-matured DCs loaded with peptide also increased the expression of surface PD-1 in a peptide dose-dependent manner with a peak at 2 days after co-culture (Fig 3B). Importantly, there was no significant difference in the levels of PD-1 on T cells co-cultured with polyI:C and LPS pre-treated DCs (Fig 3B). Blockade of PD-L1 using anti-PD-L1 blocking antibodies increased T cell proliferation when DCs were not matured or matured with polyI:C (Fig 3C). Interestingly, no effect of PD-L1 blockade on T cell proliferation was observed when T cells were co-cultured with LPS-matured DCs even at low peptide concentrations (Fig 3C). This selective effect of PD-L1 on T cell proliferation restricted to DCs matured with polyI:C and not LPS was even more pronounced when we used DCs from PD-L1-deficient mice (Fig 3D). These results suggest that the low T cell proliferation observed upon polyI:C-induced maturation of DCs is, in part, due to the expression of PD-L1 on DCs.

### PD-L1 ligand trafficking is selectively modulated upon polyI:C treatment

The unexpected observation that T cell proliferation induced by polyI:C-, but not LPS-treated DCs, was susceptible to PD-L1 blockade led us to question whether PD-L1 itself was regulated differently between the two DC populations. Indeed, in the absence of T cells, PD-L1 expression on the DCs were comparable between both treatments. We questioned whether the presence of a T cell-dependent signal would modulate this PD-L1 surface expression. The interaction of CD40L on T cells with CD40 on DCs is required to induce complete DC maturation and T cell proliferation [27]. We found that CD40L on OT1 T cells increased progressively upon co-culture with peptide-loaded DCs pre-treated or not with polyI:C or LPS (S3A Fig). Expression of CD40L on T cells followed a similar kinetic to PD-1 expression on T cells with a peak at 2 days after T-DC co-culture (compare S3A Fig and Fig 3B).

**Fig 4:**
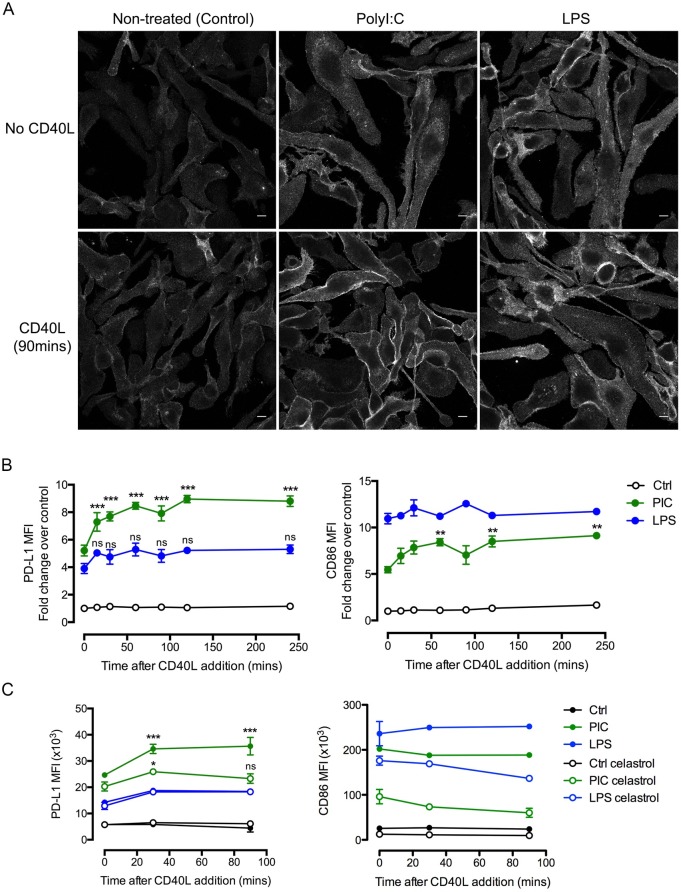
CD40L signalling induces the re-distribution of PD-L1 to the cell surface in polyI:C-treated DCs. (**A**) PD-L1 expression on treated DCs. DCs were treated with nothing (Control), polyI:C (25 μg/mL) or LPS (10 ng/ml) for 20 h followed by incubation with medium alone (no CD40L) or with rCD40L-Fc (500 ng/mL) for 90 min. PD-L1 expression was analysed by confocal microscopy. Scale bar is 10μm. Images are a single confocal plane. (**B**) Translocation of PD-L1 to the surface. DCs treated as above were incubated with rCD40L-Fc for the indicated times. Surface expression of PD-L1 (left panel) and CD86 (right panel) was evaluated by flow cytometry. Data is represented as a fold change over expression values at time 0 of non-treated (Ctrl) DCs. (**C**) DCs stimulated as above were incubated with celastrol (3 μM) for 1hr before addition of rCD40L-Fc for the indicated times. Surface expression of PD-L1 (left panel) and CD86 (right panel) were evaluated by FACS. Data in B and C are a compilation of 5 and 3 independent experiments respectively. Significance is calculated for each time point as compared to time 0 of CD40L treatment. *** represents p<0.001, * p<0,05, * p<0,05 by one-way anova test. Error bars represent SEM.

In order to monitor PD-L1 trafficking alone and simplify the system, we replaced the CD40L-derived signals received from the T cell upon T-DC interactions with recombinant CD40L (rCD40L). Confocal analysis on fixed and permabilised cells showed that most of the PD-L1 is present intracellulary in control DCs with no TLR stimuli and rCD40L treatment did not significantly alter this distribution (Fig 4A). In contrast, DCs pre-treated with polyI:C but not LPS significantly increased cell surface PD-L1 after 90 min treatment with rCD40L (Fig 4A). Quantification by FACS revealed that PD-L1 surface levels of polyI:C pre-treated (but not LPS) DCs increased as early as 15 min after addition of rCD40L and peaked at 60 min suggesting together with the confocal images that this rapid cell surface increase may result from trafficking from intracellular stores rather than from a transcriptional up-regulation (Fig 4B, left panel). Of note, CD86 expression remained unchanged in control and LPS-treated DCs upon rCD40L treatment whereas it was only slightly modulated in polyI:C-treated DCs as compared to PD-L1 (Fig 4B, right panel). No prominent difference in PD-L1 location was observed upon CD40L treatment in LPS-matured DCs (Fig 4A) although CD40 expression was consistently and homogenously higher in LPS-treated DCs as compared to polyI:C treatment (S3B Fig) indicating that the CD40L-induced increase of surface PD-L1 was specific to polyI:C-treated DCs. This increase in cell surface PD-L1 was dependent on CD40 signalling since celastrol, an inhibitor for the NF-kB signalling cascade downstream of CD40, inhibited the rCD40L-induced PD-L1 trafficking in polyI:C-treated DCs while basal PD-L1 surface levels were unaffected (Fig 4C, left panel). Of note, celastrol treatment reduced basal CD86 expression but there was no effect on CD86 expression upon CD40L treatment (Fig 4C, right panel). These results indicate that PD-L1 trafficking to the cell surface upon CD40 signalling is selectively modulated in polyI:C pre-treated DCs in an NF-kB dependent manner.

### PD-L1 surface expression on polyI:C-treated DCs reduces T cell contact time

**Fig 5:**
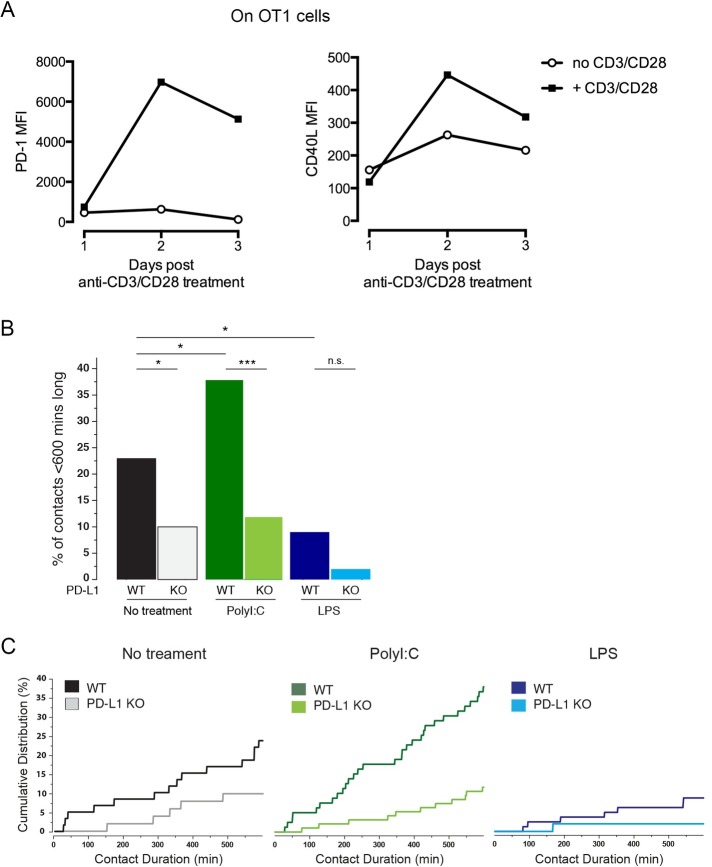
PD-L1 surface expression can regulate the DC-T time of contact. (**A**) Cell surface expression of PD-1 (left panel) and CD40L (right panel) was analysed by FACS on OT1 T cells activated with coated anti-CD3 and soluble anti-CD28 at 1, 2 and 3 days post-activation. (**B**) Time of contact between DCs and T cells. DCs from wildtype or PD-L1 KO mice were pre-treated with nothing (control), polyI:C (25 μg/mL) or LPS (10 ng/ml) and co-cultured with *Ubi*-GFP OT-I T cells pre-activated for 2 days with anti-CD3/CD28. The percentage of T cells forming contacts of <600 mins is plotted for each condition. More than 50 contacts were measured for each condition, pValues determined by Z-test (**C**) The cumulative distribution of the T-DC measured contact times. Only contact with duration <600 min are shown in the graph. ****: p<0.0001 and *: p <0.05. n.s., non-significant. Data is representative of 2 independent experiments.

Previous studies have suggested that cell surface expression of PD-1 limits the time of contact of T cells with DCs [28, 29]. Indeed, blocking of the PD-1-PD-L1 interaction between activated or tolerized T cells and PD-L1 expressing APCs leads to longer arrest of T cells on APCs and a better effector response. We thus investigated whether the low T cell response observed with polyI:C-treated DCs was due to the lack of stable DC-T cell contacts. PD-1 and CD40L surface expression were concomitantly increased on OT1 T cells when activated with anti-CD3 and CD28 Abs with a peak at day 2 after activation (Fig 5A). To measure the DC-T contact times in the various conditions, we performed 12 h imaging of co-cultures between *Ubi-*GFP OT1 T cells activated for 2 days (thus PD-1^+^CD40L^+^) and untreated, polyI:C- or LPS-treated DCs that were peptide-loaded. Analysis of the movies revealed that more than 60% of the observed contacts were longer than the duration of the recording as expected in such strong stimulation conditions. However, the proportion of contacts measured that were shorter than 600 min significantly changed between the different conditions (Fig 5B). The cumulative distributions of the measured contact times indicate that the changes in contact time can be attributed to the duration of contacts of the whole population (Fig 5C). The proportion of shorter contacts significantly increased from 23% in non-treated DCs to 38% in polyI:C-treated DCs, while it was reduced to 9% in LPS-treated DCs. This suggests that TLR3 signalling in DCs leads to the reduction of contact times with specific T cells, while TLR4 signalling extends the duration of the contacts allowing for better T cell activation (see Fig 2B). Conversely, the absence of PD-L1 on the DCs lowered the percentage of cells forming shorter contacts between T cells and DCs in all conditions. In polyI:C-treated DCs the proportion strongly decreased from 38% to 12% whereas the reduction was moderate in LPS-treated DCs although not statistically significant. Thus, presence of PD-L1 on polyI:C-treated DCs strongly impacts on DC-T cell contact duration and correlates with T cell activation. These observations are in line with the absence of a PD-L1-dependent regulation of T cell proliferation in LPS-treated DCs suggesting again that the regulation of PD-L1 expression and function in LPS- and polyI:C-treated DCs are distinct. We conclude that expression of PD-L1 at the surface of polyI:C-treated DCs is responsible for shortening the contact time with specific activated T cells.

## Discussion

TLR ligands have often been grouped together as potent activators of the innate immune response although their ligands and signalling pathways are quite distinct depending on the cell type. Certain TLR ligands are more successful as adjuvants in tumour immunotherapy as compared to others [8]. Several clinical trials with varied success are ongoing wherein polyI:C is used as an adjuvant. Nevertheless, studies on pre-clinical models strongly suggest that the timing of polyI:C administration is crucial for a potent adjuvant effect [15, 18]. Indeed, few studies have shown that systemic infection or pre-treatment of mice with TLR ligands hinders subsequent CD8^+^ T cell effector responses by impairing DC function [13, 18].

In the present study, we sought to dissect the effect of polyI:C treatment of DCs on the induction of an effector CD8^+^ T cell response. We found that although the stimulation of DCs through TLR3-specific polyI:C induced DC maturation and a potent inflammatory response, the ability to induce effector CD8+ T cell proliferation in response to the minimal OVA peptide was lower as compared to LPS. Although, total PD-L1 levels were comparable in the two types of DCs upon overnight TLR stimulation, T cell proliferation induced by polyI:C-treated and not LPS-treated DCs was negatively regulated by PD-L1. This selective PD-L1-dependence could be explained by a further increase of surface PD-L1 upon CD40 signalling specifically on polyI:C-treated DCs. To our knowledge, this is the first time that the CD40-CD40L interaction has been shown to affect the trafficking of PD-L1 in DCs.

PD-1 is actively recruited to the immune synapse if PD-L1 or PD-L2 is present on the APC [30]. However, little is known about the intracellular trafficking of PD-L1 and it is still debated whether PD-L1 itself can signal with its short cytoplasmic tail. Besides an increase of total PD-L1 expression upon DC maturation, we show here that CD40 signalling can lead to the trafficking of PD-L1 to the cell surface specifically in polyI:C-treated DCs. CD40 signalling was previously shown to regulate surface recycling of FCgRIIb in DCs [31] suggesting that this pathway may be implicated in the regulation of trafficking of a group of proteins involved in the control of adaptive immune responses.

One of the novel observations of this study is the differential programming of DCs by TLR3 and TLR4 signalling with regards to PD-L1-mediated inhibition of T cell responses. Indeed, though polyI:C and LPS-treated DCs express comparable levels of PD-L1, only polyI:C-treated DCs inhibit T cell responses through PD-L1. These results suggest that increase of PD-L1 expression upon DC maturation alone is not enough for PD-L1-mediated suppression. A second signal delivered by the T cell itself, such as the CD40-CD40L interaction, is crucial to limit the T cell response. Although cytokines secreted by the T cell might influence PD-L1 signaling, the fact that we observe a re-location of PD-L1 from intracellular pools to the cell surface upon recombinant CD40L treatment suggests that a cytokine-independent mechanism is in play. The cytoskeletal dynamics of both the T cells and DCs are altered upon the formation of an immune synapse. The rapid formation of the immunological synapse recruits several signaling molecules that govern the quality and intensity of the T cell response. We observed that the inhibition of NF-kB signaling using celastrol abrogated the re-location of PD-L1 to the cell surface upon recombinant CD40L treatment suggesting that a signaling event downstream of CD40 recruited to the immunological synapse is responsible for such PD-L1 regulation.

How a further increase of surface PD-L1 induced by CD40 signalling can influence PD-L1-mediated T-DC contact and T cell function is intriguing. Although we point out that CD40L induces PD-L1 re-location to the cells’ surface, this might not be an independent event. Other cell surface or signaling molecules resulting in the stabilization of PD-L1 or the immunological synapse might also be affected thus governing the results of the T-DC interaction. The observation that polyI:C and not LPS induces such a regulation suggests that these transcriptionally distinct cells respond differently to T cell encounter.

Chronic viral infections and tumours are often associated with high expression of PD-1 on T cells that makes them refractory to TCR stimulation upon interaction with PD-L1/L2. Besides the PD-1 ITIM-mediated suppression of TCR signalling, PD-1-PD-L1 interaction prevents stable contacts to form between DCs and T cells that are required for potent activation of the T cells [28, 29]. Indeed, we show that PD-L1 induced by polyI:C (but not LPS) treatment led to shorter contacts between T cells and DCs. From our results we infer that in this case the interaction of CD40L on the T cells with CD40 on the DCs leads to an increase in PD-L1 surface expression and therefore fewer long and stable contacts between these cells. This mechanism could explain the low proliferation and effector function of the T cells activated by polyI:C-treated DCs. It may also contribute in vivo to the limitation of the spread of the activation of antigen specific CD8 T cells in a simple manner directly linked to the success of the activation of the CD8 T cell response. To our knowledge, this is the first time that such precise regulation of PD-L1 trafficking induced by TLR signalling has been documented.

TLR3 can detect self RNA upon cellular necrosis [32, 33] suggesting that the high expression of TLR3 within certain tumours might enable detection of RNA released from necrotic tumour cells. Indeed, high ionizing radiation as therapy also leads to necrosis and the possible release of TLR3 ligands. Interestingly, PD-L1 expression increases in certain tumours after radiation therapy. The coupling of anti-PD-L1 treatment with high ionizing radiation therapy dramatically reduced the regrowth of tumours [34] suggesting again a synergistic action between detection of necrotic ligands such as RNA and PD-L1 blockade. Recently, a synergistic action of polyI:C and anti-PD-L1 blockade was demonstrated in mouse models of melanoma [23]. Interestingly, in a mouse model of tumour immunotherapy, the use of PD-L1-deficient DCs pre-activated with polyI:C increased anti-tumour T cell activity [35]. Our results showing that PD-L1 blockade is especially crucial for effective CD8^+^ T cell responses upon polyI:C treatment go hand-in-hand with these systemic studies suggesting that cancer immunotherapy using polyI:C would strongly benefit from anti-PD-L1 checkpoint blockade. We further provide a mechanistic insight for the combined effectiveness of such a treatment during tumour immunotherapy.

## Supporting Information

S1 Fig Characterisation of the TLR3-specific response to low molecular weight polyI:C. (**A**) Wildtype or TLR3 KO DCs were treated with LPS or different doses of polyI:C for 20 h and the expression of CD86 was monitored by FACS. (**B**) Cytokines secreted by wildtype or TLR3 KO DCs stimulated with nothing (Ctrl), polyI:C or LPS for 20 h were analysed in the supernatant. Data are representative of 3 independent experiments. n.d. stands for not-detected.
(TIF)

S2 Fig Titrations of polyI:C and LPS on DCs for expression of surface markers. DCs were treated with nothing (Ctrl), different doses of polyI:C or different doses of LPS for 20 h. The expression of CD86, CD80, H2K^b^ and CD40 were monitored by FACS. Data are represented as the ratio of MFI of the given antibody over its isotype control.
(TIF)

S3 Fig CD40 and CD40L expression on stimulated cells. (**A**) Surface CD40L expression on OT1 T cells co-cultured with DCs pre-treated with nothing (Ctrl), polyI:C (PIC) or LPS for 20 h and loaded with different concentrations of the SIINFEKL peptide was monitored over time by FACS. Data is representative of 2 independent experiments. (**B**) Left, FACs plots PD-L1 and CD40 co-expressed on DCs treated with polyI:C (in green) and LPS (in blue) as compared to non-treated DCs (in grey). Right, MFI of surface CD40 expression on DCs treated with nothing, polyI:C or LPS for 20 h was analysed by FACS. Each dot represents data from one independent experiment
(TIF)
