# Supplementary figures and images for: TLR3-Induced Maturation of Murine Dendritic Cells Regulates CTL Responses by Modulating PD-L1 Trafficking
Source: PLoS One. 2016 Dec 2;11(12):e0167057. doi: 10.1371/journal.pone.0167057 (PMC5135054; doi:10.1371/journal.pone.0167057)

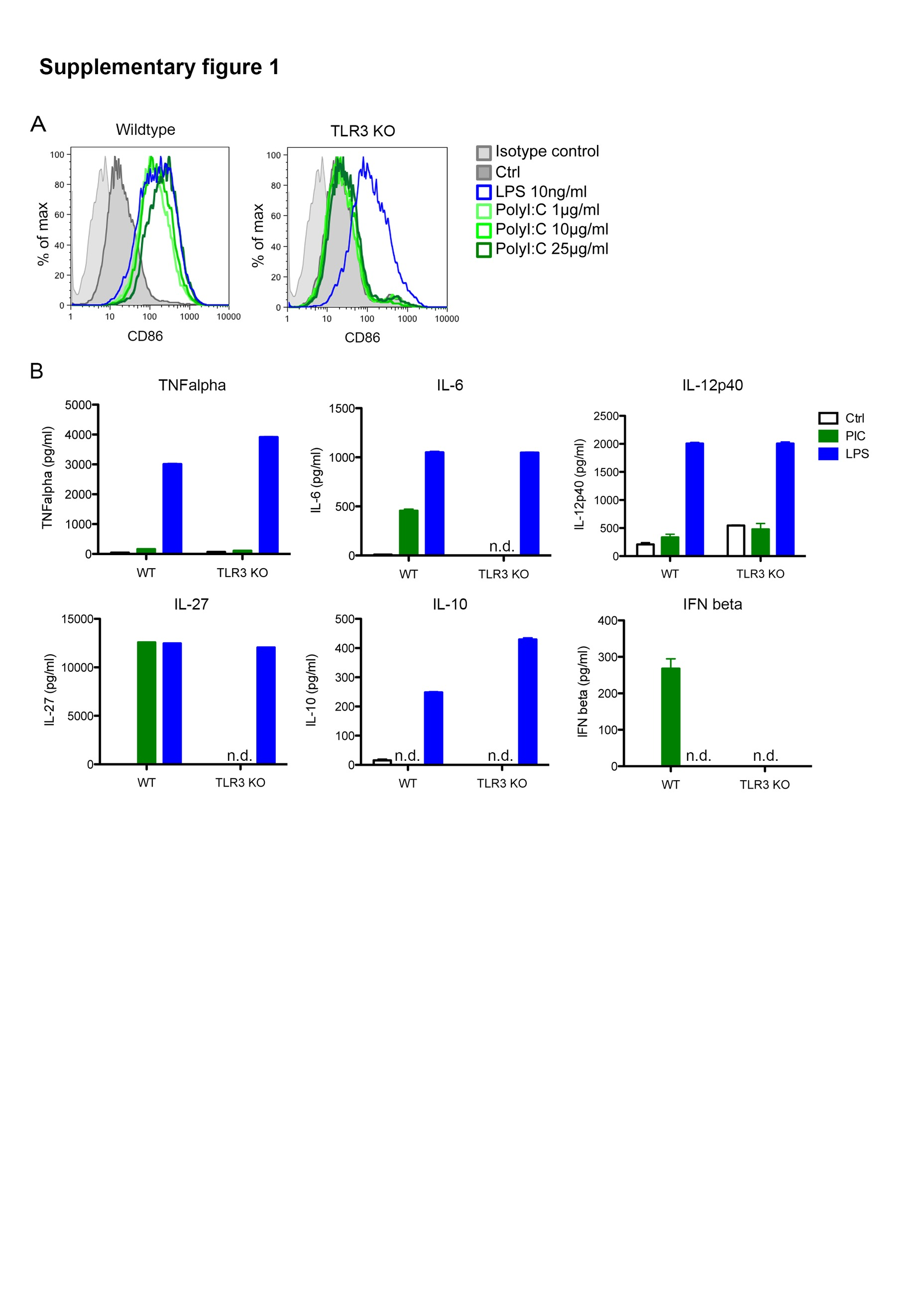

Supplement: S1 Fig — (A) Wildtype or TLR3 KO DCs were treated with LPS or different doses of polyI:C for 20 h and the expression of CD86 was monitored by FACS. (B) Cytokines secreted by wildtype or TLR3 KO DCs stimulated with nothing (Ctrl), polyI:C or LPS for 20 h were analysed in the supernatant. Data are representative of 3 independent experiments. n.d. stands for not-detected. (TIF) [file pone.0167057.s001.tif]

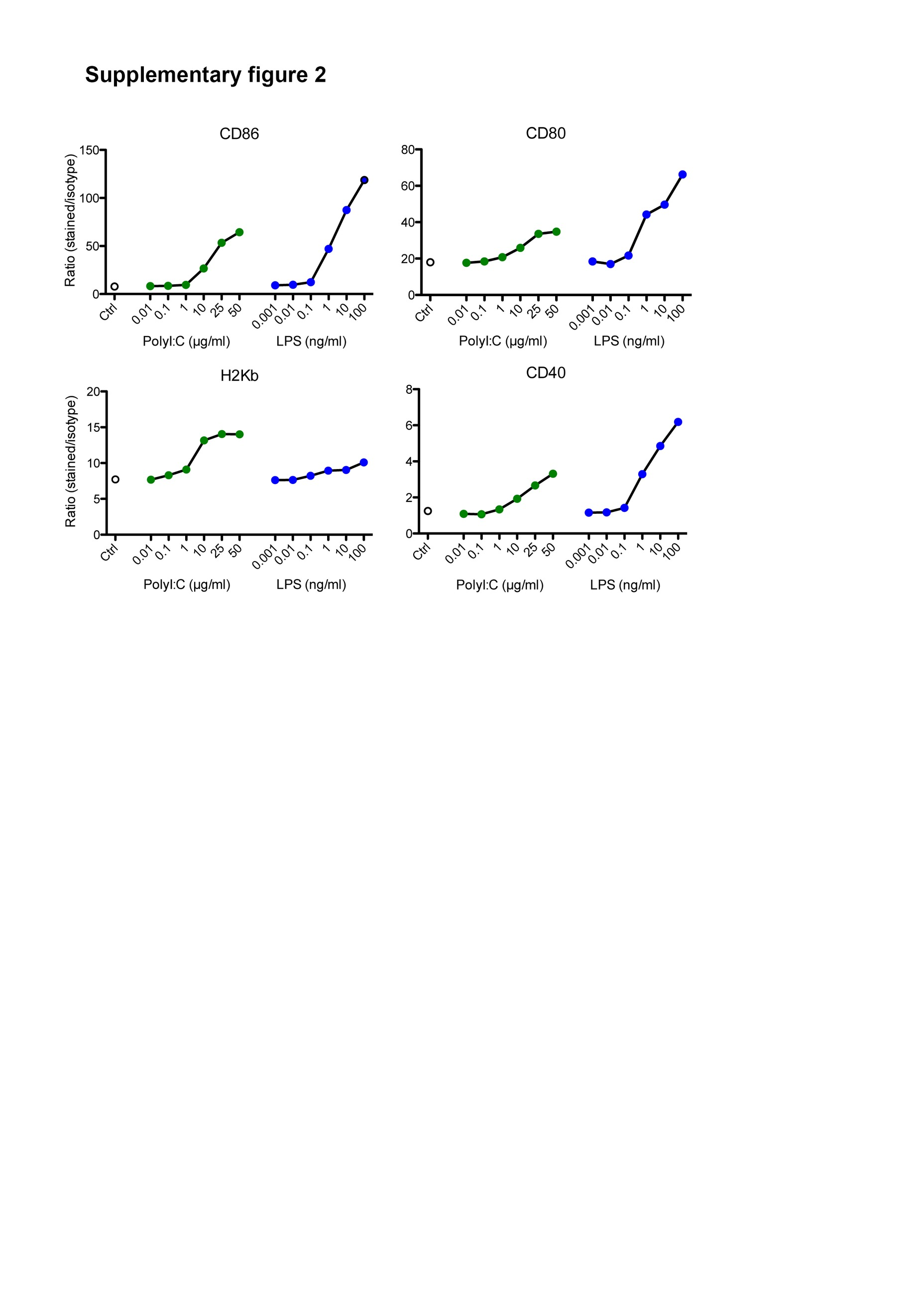

Supplement: S2 Fig — DCs were treated with nothing (Ctrl), different doses of polyI:C or different doses of LPS for 20 h. The expression of CD86, CD80, H2Kb and CD40 were monitored by FACS. Data are represented as the ratio of MFI of the given antibody over its isotype control. (TIF) [file pone.0167057.s002.tif]

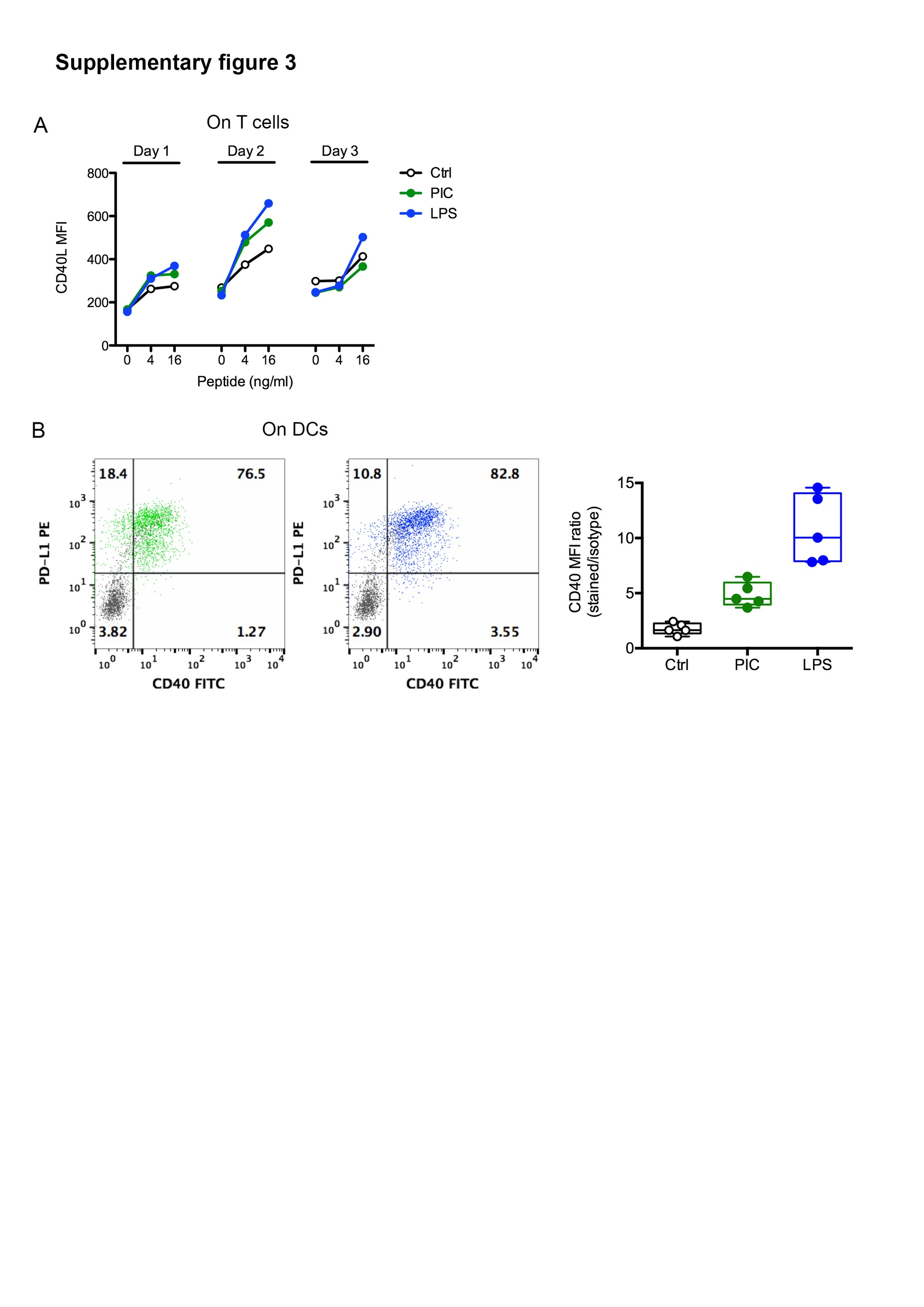

Supplement: S3 Fig — (A) Surface CD40L expression on OT1 T cells co-cultured with DCs pre-treated with nothing (Ctrl), polyI:C (PIC) or LPS for 20 h and loaded with different concentrations of the SIINFEKL peptide was monitored over time by FACS. Data is representative of 2 independent experiments. (B) Left, FACs plots PD-L1 and CD40 co-expressed on DCs treated with polyI:C (in green) and LPS (in blue) as compared to non-treated DCs (in grey). Right, MFI of surface CD40 expression on DCs treated with nothing, polyI:C or LPS for 20 h was analysed by FACS. Each dot represents data from one independent experiment (TIF) [file pone.0167057.s003.tif]
